# What about False Insights? Deconstructing the Aha! Experience along Its Multiple Dimensions for Correct and Incorrect Solutions Separately

**DOI:** 10.3389/fpsyg.2016.02077

**Published:** 2017-01-20

**Authors:** Amory H. Danek, Jennifer Wiley

**Affiliations:** Department of Psychology, University of Illinois at ChicagoChicago, IL, USA

**Keywords:** aha experience, insight, problem solving, false insights, phenomenology, suddenness, pleasure, confidence

## Abstract

The subjective Aha! experience that problem solvers often report when they find a solution has been taken as a marker for insight. If Aha! is closely linked to insightful solution processes, then theoretically, an Aha! should only be experienced when the correct solution is found. However, little work has explored whether the Aha! experience can also accompany incorrect solutions (“false insights”). Similarly, although the Aha! experience is not a unitary construct, little work has explored the different dimensions that have been proposed as its constituents. To address these gaps in the literature, 70 participants were presented with a set of difficult problems (37 magic tricks), and rated each of their solutions for Aha! as well as with regard to Suddenness in the emergence of the solution, Certainty of being correct, Surprise, Pleasure, Relief, and Drive. Solution times were also used as predictors for the Aha! experience. This study reports three main findings: First, false insights exist. Second, the Aha! experience is multidimensional and consists of the key components Pleasure, Suddenness and Certainty. Third, although Aha! experiences for correct and incorrect solutions share these three common dimensions, they are also experienced differently with regard to magnitude and quality, with correct solutions emerging faster, leading to stronger Aha! experiences, and higher ratings of Pleasure, Suddenness, and Certainty. Solution correctness proffered a slightly different emotional coloring to the Aha! experience, with the additional perception of Relief for correct solutions, and Surprise for incorrect ones. These results cast some doubt on the assumption that the occurrence of an Aha! experience can serve as a definitive signal that a true insight has taken place. On the other hand, the quantitative and qualitative differences in the experience of correct and incorrect solutions demonstrate that the Aha! experience is not a mere epiphenomenon. Strong Aha! experiences are clearly, but not exclusively linked to correct solutions.

## Introduction

Theoretically, false insights should not exist. The founders of insight research, the Gestalt psychologists, understood insight to be the result of a productive thinking process turning a problem, or “defective Gestalt,” into a solution, a “good Gestalt” (Wertheimer, 1925, 1959; Duncker, 1945). This classical view of insight as being defined by a restructuring of the problem representation (Wertheimer, 1925) implies that an insight always results in a correct solution, as for example also postulated by Sandkühler and Bhattacharya (2008). The subjective Aha! experience that problem solvers often report when they find a solution has been taken as a marker for insight (e.g., Kaplan and Simon, 1990; Gick and Lockhart, 1995) and researchers have relied on self-reports of the Aha! experience to distinguish insight solutions from non-insight solutions (e.g., Jung-Beeman et al., 2004; Kounios et al., 2006; Subramaniam et al., 2009). If the Aha! experience is closely linked to insightful solution processes based on restructuring (“representational change” in terms of Ohlsson, 1992), then theoretically, an Aha! should only be experienced when the correct solution is found (i.e., a “true insight”). This implies that the Aha! experience should be different or even non-existent for incorrect solutions. On the other hand, already Ohlsson theorized that “erroneous insights” could exist (Ohlsson, 1984b, p. 124) and that they would arise if a solution attempt that seems promising at first glance does not map onto the actual problem space. However, the question of the existence of false insights (experiences that feel like insights during incorrect solution attempts) has not received much attention so far. Empirical findings regarding the nature of Aha! experiences during false insights are sparse because incorrect solutions are typically discarded and not further analyzed. Exceptions are recent studies by Danek et al. (2014b), Salvi et al. (2016), and Webb et al. (2016) which will be discussed in detail further below.

Empirical support for the strong position that insight is linked to finding a correct solution, comes from one study by Metcalfe (1986b). She was the first to look at metacognition during problem solving by using feeling-of-warmth ratings on a set of problems thought to require insight for solution. She found that warmth ratings differed as a function of solution correctness: 76% of all correct solutions were preceded by a “subjectively catastrophic process” (Metcalfe, 1986b, p. 633), measured as a sudden increase in warmth ratings upon finding a solution (from a previous flat line). In contrast, incorrect solutions were more likely to be preceded by a gradual increase in warmth. Although her results were not completely clear-cut (52% of all incorrect solutions also showed the pattern of a sudden increase), this initial study provided evidence that the subjective perception of solutions as sudden may be linked to correctness. However, although subjective perceptions were assessed with feelings-of-warmth in this study, participants' subjective Aha! experiences were not.

Three more recent studies that did assess participants' subjective Aha! experiences using self-reports have found a small percentage of false insights, i.e., Aha! experiences that were reported for incorrect solutions (Danek et al., 2014b; Hedne et al., 2016; Salvi et al., 2016). Apart from trial-wise Aha! ratings, these studies did not examine the Aha! experience any further, so it remains an open question whether Aha! experiences reported after incorrect solutions differ from those reported after correct solutions. There is some evidence from a study by Sandkühler and Bhattacharya (2008) that correct solutions are processed differently than incorrect solutions with stronger gamma band activity (40 Hz) over parieto-occipital regions. Interestingly, Jung-Beeman et al. (2004) also reported a sudden burst of gamma band activity in the right anterior superior temporal gyrus about 0.3 s prior to solution (only for insight solutions as compared to non-insight solutions). Further, Salvi et al. (2016) found that Aha! experiences are more likely to be reported following correct solutions than incorrect ones. Similarly, but without splitting their analysis into correct and incorrect solutions, Webb et al. (2016, reported in the same Research Topic) found that a feeling of Aha! is positively associated with accuracy. Finally, it is important to note that in all of these studies (and in the present study, too), problem solvers did not receive any feedback about the correctness of their solutions which suggests that possible differences in the Aha! experience between correct and incorrect solutions were not due to solvers' awareness that they had suggested an incorrect solution. The aim of the present study was to more directly compare whether differences might be found in subjective Aha! experiences for correct vs. incorrect solutions.

## Defining the dimensions of Aha!

The Aha! experience is probably not a unitary construct, but has several different facets. This is reflected in the following typical instruction given to participants as part of self-report methods:

A feeling of insight is a kind of “Aha!” characterized by suddenness and obviousness. You may not be sure how you came up with the answer, but are relatively confident that it is correct without having to mentally check it. It is as though the answer came into mind all at once—when you first thought of the word, you simply knew it was the answer. This feeling does not have to be overwhelming, but should resemble what was just described. (Jung-Beeman et al., 2004, p. 507).

Such definitions of an Aha! generally include many different dimensions of experience which clouds the interpretation of which dimensions are most important. In Jung-Beeman's Aha! prompt, the dimension of Suddenness in the emergence of the solution is described (literally, and also by “all at once”), as well as a feeling of Obviousness and Certainty (which both seem to refer to the same sensation, namely being sure about the correctness of a solution). Then there is the additional aspect of not having used a clear strategy (“You may not be sure how you came up with the answer” and “You simply knew it was the answer”). Other researchers focus on different dimensions, for example, based on earlier work that characterized insightful solutions as sudden and surprising (Metcalfe, 1986a,b; Metcalfe and Wiebe, 1987; Schooler et al., 1993; Davidson, 1995; Bowden, 1997), Cushen and Wiley (2012) used the following prompt: “If you figured out how to solve the puzzle, how surprised were you? How much did it feel like a sudden realization?” relying on only two dimensions, Suddenness and Surprise, to characterize the Aha! experience. There is no consensus about which components make up the Aha! experience which unfortunately leads to a large variety in which dimensions are used across studies. In fact, with every research group creating their own definition of Aha! experiences, it is nearly impossible to find studies that use the same prompts. Therefore, a systematic analysis of how much each purported dimension predicts the overall Aha! experience would be useful.

**Figure 1:**
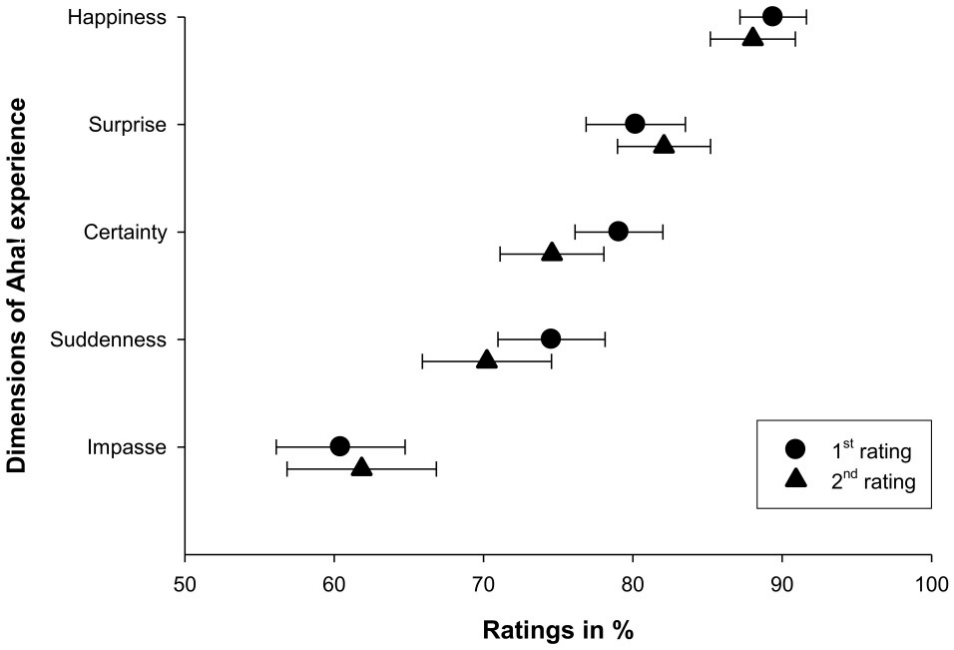
**Ratings of importance on a visual analog scale from 0 to 100**. Ratings were repeated after a 14 days delay. Figure as originally published in Danek et al. (2014a).

A main goal of this study was to decompose the Aha! experience along its different dimensions in order to identify those dimensions that best predict a global Aha! rating. This would then allow for the investigation of which dimensions might differ in their relation to correct and incorrect solutions. Danek et al. (2014a) provided an initial attempt to determine which specific dimensions drive the Aha! experience. In this study, participants attempted to discover solutions to a set of magic tricks (a task which has been demonstrated to lead to Aha! experiences; Danek et al., 2013, 2014b). At the end of the study, participants were asked to think back to the Aha! experiences they had during the study, describe them in an open-ended response, and rate the importance of several individual dimensions. As shown in Figure 1, high endorsement implicated the dimensions of Happiness, Surprise, Certainty and Suddenness as important for the Aha! experience both at the end of the study (1st rating) and after 14 days (2nd rating). Open-ended responses also suggested Drive (being motivated to continue problem solving) and Relief (feeling relieved or relaxed) as two further dimensions. However, these data were collected only once at the end of the study, which means they could not be used to align performance and Aha! experiences on particular problems. In contrast, the present study will take trial-wise ratings after each solution attempt. Just recently, the same approach was chosen by Webb et al. (2016) who had participants solve sets of insight and non-insight problems and collected trial-wise ratings of Certainty (“Confidence” in their study), Pleasure, Surprise and Impasse along with a measure of the intensity of the insight experience (“Strength”), using the same visual analog scales as Danek et al. (2014a, namely a continuous scale from 0 to 100) that allow a more fine-grained assessment of these feelings than the typically used binary or Likert scales.

In the present study, after each trick, participants were asked to rate six dimensions of their solution experience, based on prior work and intended to represent both cognitive and affective dimensions. Each dimension is illustrated by a short quotation from participants' open-ended descriptions of “What an Aha! moment feels like” in Danek et al.'s study (Danek et al., 2014a).

**Suddenness**. Cognitive dimension. “The moment comes quite suddenly, as if the idea jumps directly into your mind and doesn't develop step by step by reflection.”

That an insightful solution appears suddenly rather than incrementally is thought to be a key characteristic of insight, consistent with the findings of Metcalfe (1986b) and Metcalfe and Wiebe (1987) who demonstrated a discontinuous pattern of feeling-of-warmth ratings. The Gestalt psychologists encompassed the idea of Suddenness of insight in their writings (e.g., Duncker, 1945). This idea was further corroborated by Davidson (1995) and also by Sandkühler and Bhattacharya (2008) who reported high ratings of Suddenness for correct solutions.

**Certainty**. Cognitive dimension. “A feeling of definite knowledge or alternatively, a first sensation of knowledge that is not necessarily confirmed in the next step, but initially, feels certain and irrefutable.”

The obviousness of insightful solutions, or the “intuitive sense of success” (Gick and Lockhart, 1995, p. 215) was emphasized as an important aspect of the Aha! experience by Jung-Beeman et al. (2004) and is also apparent in anecdotal reports of scientific discoveries (Irvine, 2015). By separately asking for a confidence rating and an Aha! rating after each solution, Danek et al. (2014b, Experiment 1) found that participants were more confident in the correctness of their Aha! solutions than in the correctness of their non-Aha! solutions. Hedne et al. (2016, reported in the same Research Topic as the present study) just recently replicated this effect (higher confidence about insight solutions compared to non-insight solutions) in a study using a very similar set of magic tricks (see Supplementary Material for full trick list).

**Pleasure**. Affective dimension. “I feel lively and happy to have figured it out. A feeling of bliss.”

This dimension was included because problem solvers endorsed having pleasant feelings after a solution (“Happiness”) stronger than any other dimension in Danek's previous study (Danek et al., 2014a). Based on this finding, it was predicted that Pleasure would be the strongest predictor of the global Aha! rating. Of course, the emotional reaction to gaining an insight can also be negative. Already Wertheimer has described the example of a lawyer who suddenly realizes that he has burnt important documents (Wertheimer, 1925, p. 173). Gick and Lockhart (1995, p. 199) also pointed out the “groan response” or “feeling of chagrin” that sometimes comes with gaining insight, and recently, Hill et al. found evidence for such “Uh-oh moments” in reports of everyday insight experiences in an online study (Hill and Kemp, 2016). The negative aspect of insight was included in the present study with the scale for Pleasure going from “unpleasant” to “pleasant,” but not as an individual dimension.

**Surprise**. Affective dimension. “I feel surprised that I have understood something.”

An insight is often thought to feel surprising, and Gick and Lockhart (1995) suggested that surprise might constitute one of the main components of Aha! experience. However, empirical evidence for this dimension is lacking with the exception of our previous study, where Surprise was endorsed significantly less than Happiness (Danek et al., 2014a), but on the same level as Certainty and Suddenness.

**Relief**. Affective dimension. “It was a feeling of relief combined with a feeling of happiness after a phase of strain caused by failure.”

The idea that tension is released or that some kind of relaxation comes about with insight already figures in the Gestalt concept of insight (Duncker, 1945), as also noted by Ormerod et al. (2002). Relief could also reflect the overcoming of an impasse (see below), and therefore be a marker for the underlying representational change processes leading to correct solutions. Empirically, first evidence for this dimension came from open-ended questions about how an Aha! moment feels like (Danek et al., 2014a) where problem solvers repeatedly described feelings of relaxation and relief.

**Drive**. Affective dimension. “This feeling gives me wings that make me continue working on the problem which I had not been able to solve before. And, naturally, I immediately feel inclined to solve further problems, as it seems now you can do anything, no matter which task you have been set.”

This is another new dimension that was derived from open-ended questions in a prior study on the same stimulus set (Danek et al., 2014a) and that has already been described on a theoretical level (as an “energizing effect on problem solving behavior” Ohlsson, 1984a, p. 70).

### Excluded dimensions

For the sake of completeness, further possible dimensions of the multi-faceted Aha! experience are listed here, together with an explanation why they were not included in the present study.

#### Impasse

A feeling of being stuck. This dimension was rated significantly lower than all other dimensions in Danek et al. (2014a), with ratings near the midline. Further, in Webb et al. (2016), impasse was shown to be negatively correlated to the strength of self-reported Aha! experiences which supports the idea that although impasse might be part of the problem solving process, it is not part of the Aha! experience itself. Being in an impasse would also happen at a different point in time, namely before a solution is found.

#### Feelings of frustration

As discussed above, by implementing the dimension Pleasure with the two poles “unpleasant” and “pleasant,” a strong negative affective reaction is already contained in the Pleasure scale. Note that participants only see the scale with the two anchors, but not the title “Pleasure.”

#### Processing fluency

Topolinski and Reber (2010a) have argued that fluency (in the sense of a certain ease of thinking, when thoughts flow uninterruptedly and smoothly) might be the overarching feature of the Aha! experience, the “glue between its experiential features” (Topolinski and Reber, 2010a, p. 404). However, for the present purpose of regressing the Aha! experience on several dimensions (and avoiding multicollinearity between predictors), this aspect seemed already sufficiently captured by the Suddenness scale. In addition, while Topolinski and Reber (2010b) used an indirect way of assessing fluency (by varying the onset of shown solutions) that was not feasible within the present paradigm of self-generated solutions, self-reports on processing fluency seemed rather difficult to obtain.

### Overview of the present study

The present study aimed at identifying those dimensions that best predict a global Aha! rating specifically for correct solutions by using a large problem set from the domain of magic as problem solving task (Danek et al., 2014b) and asking participants to provide a solution, a global Aha! rating, and ratings on each of the six dimensions following each trick (i.e., trial-wise). Based on Danek et al. (2014b) and Salvi et al. (2016), it was predicted that correctly solved problems should be more likely to be accompanied by Aha! experiences than incorrectly solved problems. To the extent that longer solution times are due to the use of analytic or incremental solution processes, then Aha! experiences could also be predicted to be more likely to accompany faster correct solutions. Further, if Aha! experiences are a marker for true insight, then there should be some distinction between the Aha! experiences that accompany correct solutions and incorrect solutions. Theoretically, one would expect that the thinking processes leading to incorrect solutions should be fundamentally different than those leading to correct solutions that involve representational change. However, if no quantitative or qualitative differences are found, this would suggest that the Aha! experience might be epiphenomenal rather than a defining characteristic, as some researchers have argued (e.g., Weisberg and Alba, 1981). One reason why the Aha! experience might be better considered as epiphenomenal is because problem solvers do not seem to have reliable access to their solution processes and thus cannot report on them (Ash et al., 2009). However, while it is true that several studies (e.g., Cushen and Wiley, 2012) have found a disconnect between the actual solution process and solvers' reportable experience of it, this might also simply be due to using incomplete prompts (e.g., missing important dimensions or stressing less important ones) about what an Aha! experience feels like. The present systematic dissection of Aha! will hopefully contribute to getting a clearer picture about this.

## Methods

### Participants

Participants were 70 undergraduate students from the University of Illinois at Chicago Introduction to Psychology Subject Pool who received course credit for their participation (*M* = 19.6 (*SD* = 2.8) years of age; 22 males, 48 females). All of them were tested individually. Two additional subjects were tested, but could not be included in the analysis for failing to follow the instructions. In addition, on an individual trick level, whenever a participant had pressed the solution button without typing in an answer, their ratings were not analyzed, but treated as missing values. There were 35 participants in each of two conditions that counterbalanced the direction of the individual dimension ratings. Note that all participants solved at least three tricks correctly.

### Stimuli

#### Magic tricks

A set of 37 magic tricks (listed in the Supplementary Material) were presented to participants as a problem solving task using a paradigm established by Danek et al. (2014b). Students were told “Your task is to solve this puzzle and try to see through the magic trick.” This large set of problems was used in order to generate many repeated solution events (with or without insight) that participants could report on. Short video clips (duration ranged from 6.3 to 72.5 s) were presented on a 19″ computer screen through PsychoPy (Peirce, 2007). The tricks had been performed by a professional magician, Thomas Fraps (Abbott, 2005), and recorded in a standardized theatre setting (see https://www.youtube.com/watch?v=3B6ZxNROuNw for an example clip from the set). The stimulus set covered a wide range of different magic effects (e.g., transposition, restoration, vanish) and methods (e.g., misdirection, gimmicks, optical illusions) (for more details, see Danek et al., 2014b). Two additional tricks were used for practice trials. Two of the 37 tricks were not solved by anyone and therefore not included in any analyses, resulting in a final problem set of 35 magic tricks.

#### Rating scale for global Aha! rating

**Figure 2:**
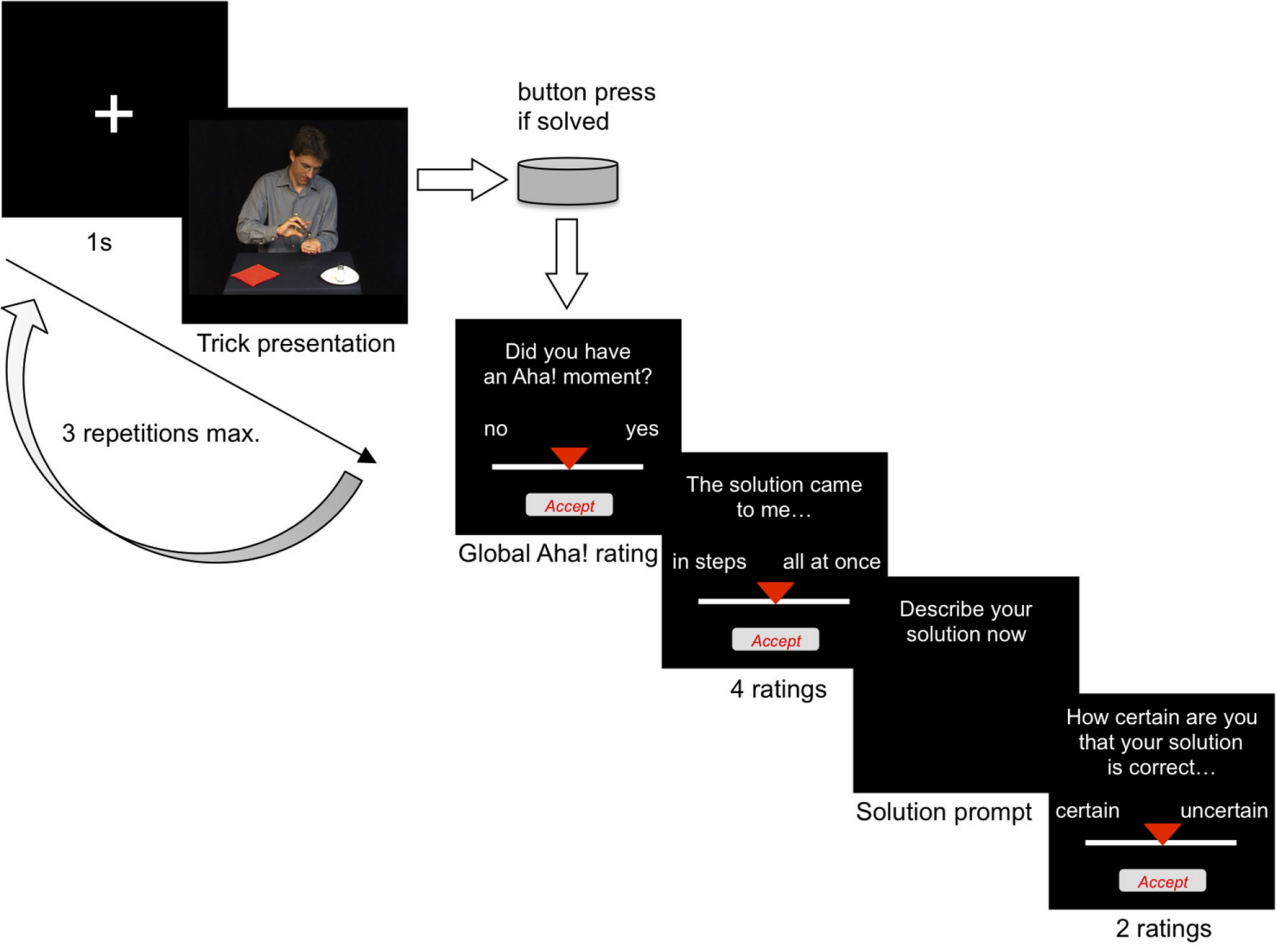
**Sequence of one trial**.

Immediately after indicating that they had found a solution, participants were asked “Did you have an Aha! moment?” and gave an answer by selecting a point between “no” and “yes” on a visual analog scale, see Slide 3 on Figure 2. In previous work, self-reports of Aha! experiences have varied between dichotomous measures (Yes - No), to Likert scales with 3, 5, or 7 points, to continuous scales. We agree with Webb et al. (2016) that binary ratings suffer from the problem that participants might use very different benchmarks for what constitutes an Aha! experience or not. Some might set the criterion for when they rate “Aha!” very high, others very low. Continuous scales allow participants to report a range of stronger and weaker Aha! experiences. Thus, the present study employed a continuous scale.

For the global Aha! scale, the “yes” anchor always appeared on the right-hand side of the scale. Participants were instructed to base their rating decision on the following description of what an Aha! moment typically feels like (translated with minor modifications from the German instruction of Danek et al., 2013; which had been originally adapted from Jung-Beeman et al., 2004):

“An Aha! moment is when the solution suddenly dawns on you and everything is clear immediately. << Experimenter snaps fingers. >> In a flash. You are relatively confident that your solution is correct without having to check it once more. In contrast, if the solution occurs to you slowly and in steps, and if you feel you still need to check it that would not be an Aha!. As an example, imagine a light bulb that is switched on all at once in contrast to slowly turning up the lights. Have you ever experienced an Aha! moment, perhaps during studying? For each solution, we ask for your subjective rating whether it felt like an Aha! moment or not. There is no right or wrong answer. Just follow your intuition.”

#### Rating scales for individual dimensions of solution experience

For each trick, participants rated their subjective solution experiences with respect to six different dimensions, using visual analog scales with the following wording for the prompts and anchors:

1. Pleasure: “At the moment of solution, my feelings were…(unpleasant - pleasant).”
2. Surprise: “The moment of solution was…(not surprising - surprising).”
3. Suddenness: “This solution came to me…(in steps - all at once).”
4. Relief: “At the moment of solution, I felt…(tense - relieved).”
5. Certainty: “How certain are you that your solution is correct: (uncertain - certain).”
6. Drive: “I am looking forward to the next trick…(no - yes).”

The dimensions appeared in the order shown above for all participants. The direction of the anchors was counterbalanced across two groups of participants. For one half of the participants, the anchors of the Pleasure, Suddenness and Drive scales were reversed from the direction of the global Aha! rating [e.g., Pleasure: “At the moment of solution, my feelings were…(pleasant - unpleasant)”]. For the other half of the participants, the anchors of the remaining three scales (Surprise, Relief, Certainty) were reversed. This created the two counterbalancing conditions.

### Procedure

After signing an agreement form, participants were seated at a computer and instructed to watch the video clips and try to find the solution. It was stressed that they should only provide plausible solutions (nothing like “a magic powder lets the coin disappear”), but that if they had an idea what the solution could be, then they should type it in even if they were not sure about it. The latter was intended to help increase the low solution rates from previous studies and generate more events of interest. They were also told to press the space bar as soon as possible once they had a solution idea. This ended the video clip presentation and brought them to the first rating screen with the global Aha! rating (see Figure 2 for the sequence of one trial). The global rating was followed by four more ratings (Pleasure, Surprise, Suddenness, and Relief). Then participants were prompted to type in their solution and finished the trial with two more ratings (Certainty and Drive). Participants did not receive any feedback on the correctness of their solutions. The procedure began with two practice trials. Then, the 37 experimental video clips were presented in randomized order. Each trick was shown a maximum of three times. If no button was pressed to indicate that a solution was found, the next trick followed. At the end of the experiment, participants filled in a demographic data sheet and were debriefed. The entire experiment lasted about 1 h.

### Response coding

Responses were coded as correct or incorrect solutions by two independent raters using a solution coding manual based on prior work with this problem set (Danek et al., 2013, 2014a,b). Correct solutions were either the real solution (i.e., the method that the magician used) or alternative, but plausible solutions, while incorrect solutions were either implausible or partial (key solution element missing) solutions. The intraclass correlation coefficient was 0.83 indicating a satisfactory level of agreement between the two raters. Conflicting cases between the two raters were resolved by a third rater.

All rating scales including the global Aha! rating were measured in whole values from 0 to 100. Solution time was measured in milliseconds from the start of the video clip until participants pressed a button to indicate that they had found a solution. Previous viewings of the trick were included in the solution times for each trial.

## Results

In total, 70 participants being presented with 35 tricks yielded 2450 observations. Of those, 603 were not solved (i.e., timeouts) and thus discarded, and an additional 69 observations were missing values due to computer errors or skipped trials. All analyses were based on the remaining 1778 observations where participants suggested a solution. Of these 1778 observations, 36.8% (654 occurrences) were correctly solved, and 63.2% (1124) were incorrectly solved. For all analyses, data were collapsed across the two counterbalancing conditions. The dataset of the present study will be made available at the open repository for psychology data “PsychData” (https://www.psychdata.de/index.php?main=none&sub=none&lang=eng).

### Relationship between solution success, solution times, and Aha! ratings

Before exploring the dimensions that predicted Aha! experiences, basic differences in the magnitudes of Aha! ratings and solution times were explored for correct and incorrect solutions.

**Figure 3:**
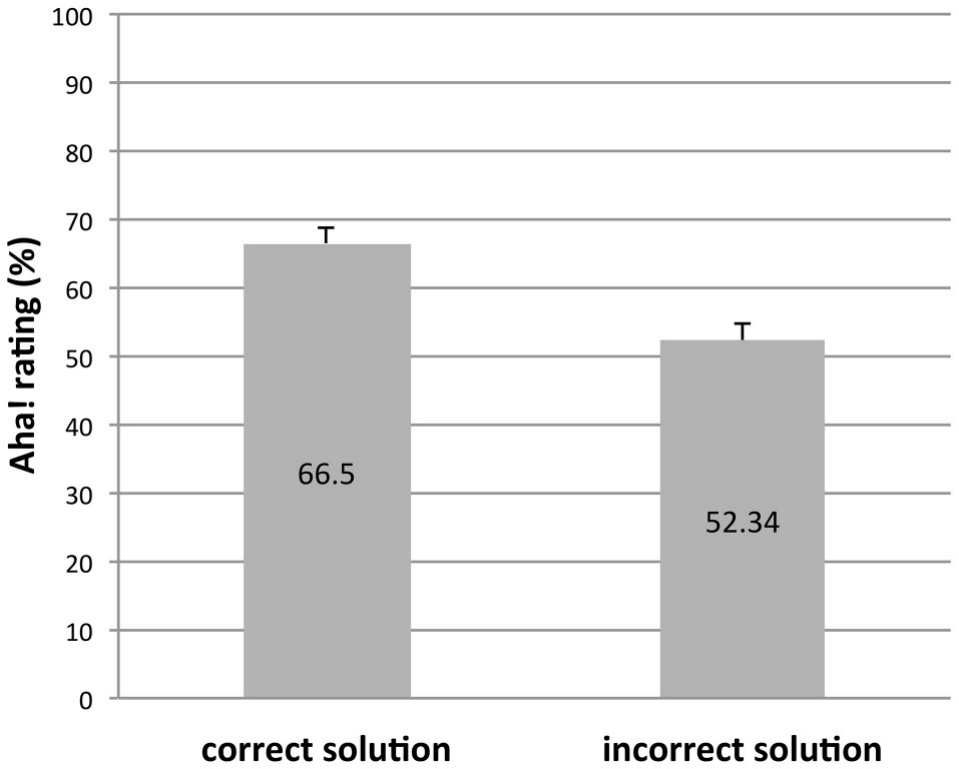
**Mean Aha! ratings as a function of solution correctness**. Error bars denote SEM.

Computing average ratings for correct and incorrect solutions at the participant level revealed that correct solutions led to higher Aha! ratings (*M* = 66.50, *SD* = 18.42) than did incorrect solutions, (*M* = 52.34, *SD* = 18.78, *t*_(69)_ = 10.21, *p* < 0.01), replicating Danek et al. (2014b) and Salvi et al. (2016), see Figure 3. This difference in the magnitude of Aha! ratings offers initial support for the position that Aha! experiences might differ following correct vs. incorrect solutions.

However, it is notable that a substantial percentage of incorrect solutions (37% or 417 out of 1124) received Aha! ratings that were higher than the average for correct solutions. This shows that the Aha! experience is not an exclusive feature of correct solutions, but that it is also reported for incorrect solutions.

**Figure 4:**
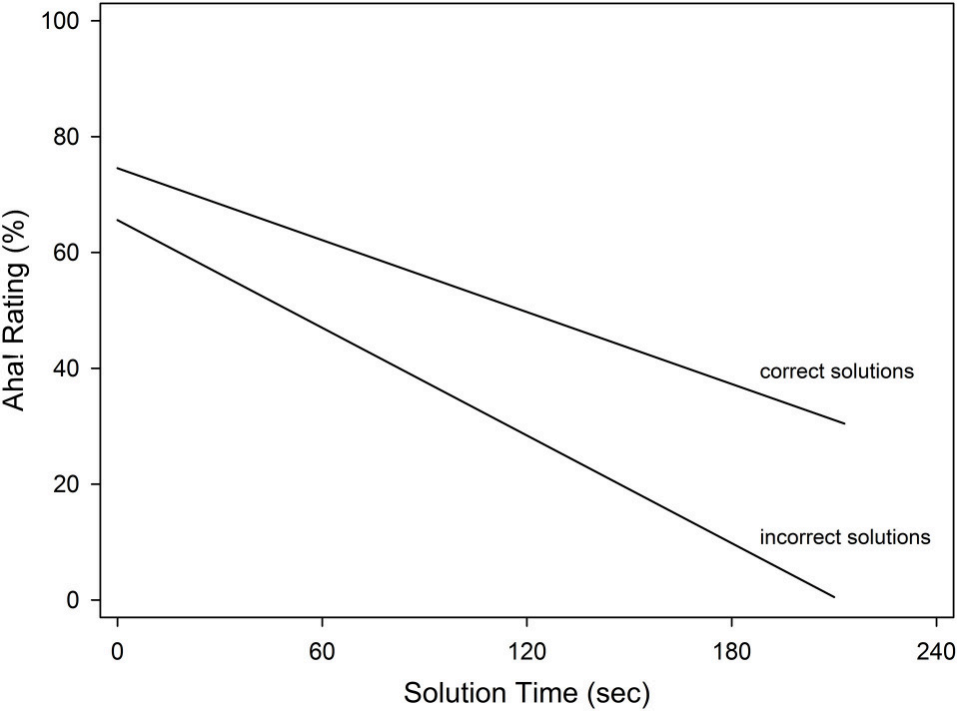
**Regression lines from the linear mixed-effects model**.

In terms of solution time, on average, correct solutions (*M* = 35.81, *SD* = 19.71) were significantly faster than incorrect solutions, (*M* = 42.46, *SD* = 24.49, *t*_(1776)_ = 6.26, *p* < 0.01). To understand the relation of solution time to Aha! ratings, a linear mixed-effects model was calculated to predict Aha! ratings, including solution time, solution correctness, and their interaction as fixed effects, and random intercepts for subjects. As shown in Figure 4, there was a main effect of solution time (*t* = 4.03, *p* < 0.01), with faster solutions more likely to be rated high on Aha! and longer solutions more likely to be rated low. There was also a main effect of solution correctness (*t* = −3.36, *p* < 0.01) with correct solutions more likely to be rated high on Aha! than incorrect solutions, as already reported above. The interaction was not significant (*t* = 1.58, *p* < 0.12). For fast incorrect solutions, it is possible that solution time is being misused as a cue because it leads to giving high Aha! ratings (“false insights”). But for longer incorrect solutions, problem solvers give low Aha! ratings, so they seem to realize that these are not “true insights.”

### Which dimensions of Aha! predict global Aha?

The main aim of the present study was to test whether differences might be found in subjective Aha! experiences after correct vs. incorrect solutions. However, before proceeding to analyses that consider only correct or incorrect solutions, we first report correlations using Webb et al.'s approach (Webb et al., 2016) of analyzing both correct and incorrect solutions together, see Table 1. We find rather similar results to theirs, with all dimensions showing a relation with Aha! ratings in simple correlations, except for the Surprise dimension. Even though the relation was still significant, we find a much lower correlation between Surprise and the global Aha! rating (*r* = 0.07, Webb et al. ranging from 0.29 to 0.48). The dimensions Suddenness, Relief and Drive were assessed only in the present study and therefore not compared with Webb et al.'s results.

**Table 1:** **Both correct and incorrect solutions: Simple correlations between participants' ratings of their problem solving experience (on the dimensions pleasure, surprise, suddenness, relief, certainty and drive) and one global Aha! rating**.

**Dimension**	**Aha! rating**	**Pleasure**	**Surprise**	**Suddenness**	**Relief**	**Certainty**	**Drive**
Aha! rating	–	0.66^**^	0.07^**^	0.49^**^	0.49^**^	0.58^**^	0.28^**^
Pleasure		–	0.10^**^	0.45^**^	0.64^**^	0.54^**^	0.34^**^
Surprise			–	−0.08^**^	0.04	−0.10^**^	0.12^**^
Suddenness				–	0.39^**^	0.41^**^	0.14^**^
Relief					–	0.53^**^	0.20^**^
Certainty						–	0.19^**^

*Dimensions listed in the order that they were asked*.

N = 1778. All values are Pearson correlation coefficients. ^*^p < 0.05.

** *p < 0.01 (2-tailed)*.

### What predicts Aha! for correct solutions?

One of the main questions for this study was which dimensions of the Aha! experience specifically predict global Aha! ratings for correct solutions. As shown in Table 2, simple correlations showed that all dimensions but Surprise were significantly and positively correlated with the global Aha! rating for tricks with correct solutions.

**Table 2:** **Correct solutions: Simple correlations between participants' ratings of their problem solving experience (on the dimensions pleasure, surprise, suddenness, relief, certainty and drive) and one global Aha! rating**.

**Dimension**	**Aha! rating**	**Pleasure**	**Surprise**	**Suddenness**	**Relief**	**Certainty**	**Drive**
Aha! rating	–	0.62^**^	0.06	0.49^**^	0.49^**^	0.52^**^	0.26^**^
Pleasure		–	0.11^**^	0.42^**^	0.68^**^	0.53^**^	0.37^**^
Surprise			–	−0.06	0.06	−0.10^*^	0.11^*^
Suddenness				–	0.35^**^	0.37^**^	0.12^**^
Relief					–	0.50^**^	0.26^**^
Certainty						–	0.20^**^

*Dimensions listed in the order that they were asked*.

N = 654. All values are Pearson correlation coefficients.

* p < 0.05.

** *p < 0.01 (2-tailed)*.

Correlations between the six dimensions and the global Aha! rating were also computed for each individual and averaged across individuals. As shown in Table 3, this led to the same pattern of results as the simple correlations. Average correlations were significantly greater than 0 for all dimensions except Surprise.

**Table 3:** **Average intra-individual correlations between dimensions and Aha! Ratings**.

**Dimension**	**Pleasure**	**Surprise**	**Suddenness**	**Relief**	**Certainty**	**Drive**
Correct	0.49^**^	0.05	0.41^**^	0.43^**^	0.48^**^	0.16^**^
Incorrect	0.61^**^	0.05	0.48^**^	0.44^**^	0.54^**^	0.18^**^

N = 68.

** *p < 0.01 one tailed t-test vs. 0. All values are mean correlations (i.e., the average of 68 individual correlation coefficients)*.

To understand the relation of each dimension to the Aha! ratings, a linear mixed-effects model was calculated to predict the Aha! ratings for just the correct solutions, including each of the dimensions as fixed effects, and random intercepts for subjects. As shown in Table 4, Pleasure, Suddenness, Certainty and Relief were found to be unique predictors of the Aha! experience for correct solutions.

**Table 4:** **Linear mixed-effects model of predictors of the global Aha! rating, for correct solutions only**.

	**Unstandardized coefficient B**	***SE B***	**β**	***P***
Constant	−3.44	4.1		
Pleasure	0.47	0.06	0.35	*p* < 0.01
Surprise	0.06	0.03	0.05	*p* = 0.061
Suddenness	0.25	0.03	0.24	*p* < 0.01
Relief	0.12	0.05	0.09	*p* < 0.05
Certainty	0.23	0.04	0.20	*p* < 0.01
Drive	−0.03	0.04	−0.03	*p* = 0.421

*N = 654*.

### What predicts Aha! for incorrect solutions?

As shown in Table 5, simple correlations showed that all dimensions were significantly and positively correlated with the global Aha! rating for tricks with incorrect solutions. However, when correlations were computed for each individual and averaged as shown in Table 3, the average correlation for Surprise was not significantly greater than 0.

**Table 5:** **Incorrect solutions: Simple correlations between participants' ratings of their problem solving experience (on the dimensions pleasure, surprise, suddenness, relief, certainty and drive) and one global Aha! rating**.

**Dimension**	**Aha! rating**	**Pleasure**	**Surprise**	**Suddenness**	**Relief**	**Certainty**	**Drive**
Aha! rating	–	0.65^**^	0.10^**^	0.47^**^	0.46^**^	0.56^**^	0.27^**^
Pleasure		–	0.11^**^	0.44^**^	0.60^**^	0.51^**^	0.30^**^
Surprise			–	−0.08	0.04	−0.09^**^	0.15^**^
Suddenness				–	0.39^**^	0.41^**^	0.13^**^
Relief					–	0.52^**^	0.15^**^
Certainty						–	0.15^**^

*Dimensions listed in the order that they were asked*.

N = 1124. All values are Pearson correlation coefficients. ^*^p < 0.05.

** *p < 0.01 (2-tailed)*.

To test which dimensions uniquely predicted Aha! ratings, a parallel linear mixed-effects model was calculated just for the incorrect solutions. As shown in Table 6, Pleasure, Suddenness, Certainty and Surprise were found to be unique predictors of the Aha! experience for incorrect solutions.

**Table 6:** **Linear mixed-effects model of predictors of the global Aha! rating, for incorrect solutions only**.

	**Unstandardized Coefficient B**	***SE B***	**β**	***p***
Constant	−11.81	2.98		
Pleasure	0.51	0.04	0.38	*p* < 0.01
Surprise	0.11	0.03	0.09	*p* < 0.01
Suddenness	0.17	0.03	0.16	*p* < 0.01
Relief	0.06	0.04	0.05	*p* = 0.08
Certainty	0.31	0.03	0.27	*p* < 0.01
Drive	0.00	0.03	0.00	*p* = 0.926

*N = 1124*.

### What distinguishes the Aha! experience between correct and incorrect solutions?

The above analyses demonstrated that Pleasure, Suddenness and Certainty were the key dimensions that combined to uniquely predict the Aha! experience for both correct and incorrect solutions. This means, Pleasure, Suddenness and Certainty ratings always covaried with Aha! ratings, independent of solution correctness. Further, Relief emerged as the one single dimension of the Aha! experience that was more likely for correct than incorrect solutions. On the other hand, Surprise was the dimension that predicted Aha! experiences only for incorrect solutions, and may be considered as misleading cue. These results suggest that all Aha! experiences may consist of a core of three dimensions, but that in addition, solution correctness may be associated with slightly different emotional coloring, with problem solvers feeling relieved for correct solutions, and feeling surprised for incorrect ones.

The other major difference between Aha! experiences for correct and incorrect solutions seems to be in magnitude. Although both were predicted by the Pleasure, Suddenness and Certainty dimensions, correct solutions were rated as more pleasant (*M* = 66.05, *SD* = 13.79) than incorrect (*M* = 56.67, *SD* = 15.63, *t*_(69)_ = 7.17, *p* < 0.01), more sudden (*M* = 55.68, *SD* = 18.17) than incorrect (*M* = 47.19, *SD* = 16.67, *t*_(69)_ = 5.90, *p* < 0.01), and solvers were more certain about being correct when they gave correct solutions (*M* = 70.55, *SD* = 14.15) than incorrect solutions (*M* = 56.14, *SD* = 16.0, *t*_(69)_ = 9.83, *p* < 0.01), even though they never received feedback about their solution correctness^1^.

### Differences in Aha! experiences due to solution complexity

Ohlsson postulated that the perceived suddenness of a solution might be a function of how much problem solving is needed to complete the problem after the initial representational change has taken place (Ohlsson, 1984b, 1992, 2011). He claimed that whether a solution feels sudden or not is contingent upon how many thinking steps are still required once a potential solution element is identified. If the entire remaining solution can be “seen” in the mind's eye [i.e., if it lies within the horizon of mental look-ahead, (MacGregor et al., 2001), which is limited by working memory capacity, (Ohlsson, 2011)], the problem will seem to be solved very quickly after the initial breakthrough. This leads to the following hypothesis (stated in chapter 4 of Ohlsson, 2011): If several additional steps are required to achieve the full solution after the first realization of a crucial solution element (Weisberg and Alba, 1981), then the solution will feel less sudden. This hypothesis can be tested within our task domain of magic tricks. Thus, the current problem set of 35 magic tricks was analyzed for the number of steps that each trick required for solution. Tricks that required just one realization after which the full solution should directly appear within the horizon of mental look-ahead, were coded as having a “single-step” solution (cf. Murray and Byrne, 2013). Alternatively, tricks that required several additional steps to reach a full solution after the first realization of the crucial solution element, were coded as “multi-step” solutions. The set was found to contain both single-step (*n* = 24 tricks) and multi-step (*n* = 11) solutions. Item-level analyses showed that correctly solved magic tricks with single-step solutions received higher Suddenness ratings (*M* = 55.69, *SD* = 7.50) than magic tricks with multi-step solutions (*M* = 47.10, *SD* = 10.69, *t*_(33)_ = 2.74, *p* < 0.05). This was independent of actual solution times which did not differ between the two groups of tricks. This analysis was computed using data for correct solutions only (because incorrect solutions vary individually and can be single- or multi-step for the same problem). Single-step solutions did not differ from multi-step solutions in any other dimension nor in the global Aha! rating nor in solution time. In contrast to Murray and Byrne's study (Murray and Byrne, 2013), single-step tricks did not differ from multi-step tricks with regard to their difficulty (measured as mean solution rate for each trick).

## Discussion

The starting point for the present study was the question whether false insights happen at all, i.e., whether high Aha! experiences are also reported for incorrect solutions. We found that overall, correct solutions were more likely to lead to Aha! experiences. However, some incorrect solutions (37%) also led to high Aha! experiences. Therefore, although the Aha! is linked to finding a correct solution, false insights clearly exist, too (as suggested by previous studies, Danek et al., 2014b; Hedne et al., 2016; Salvi et al., 2016). This shows that the Aha! experience is not an exclusive feature of correct solutions.

The present finding that correct solutions led to higher Aha! ratings than incorrect solutions is in accordance with prior studies (Danek et al., 2014b; Hedne et al., 2016; Salvi et al., 2016). Further differences were apparent with regard to solution time, with correct solutions emerging significantly faster than incorrect solutions. Both of these results offer initial support for the position that Aha! experiences might feel different for correct vs. incorrect solutions. The reasoning was if Aha! experiences are a marker for true insight, correct solutions should not only lead to higher ratings of Aha!, as found here, but also to qualitatively different ratings along the individual Aha! dimensions. If no such differences were found, this would suggest that Aha! is merely epiphenomenal, and not an indicator of different problem solving processes underlying correct and incorrect solutions.

With a systematic decomposition of the Aha! experience into its constituents, and by obtaining separate ratings for each of them, the present study found that Pleasure, Suddenness and Certainty uniquely predicted Aha! experiences for both correct and incorrect solutions. This means, when participants reported Aha!, they also had pleasant feelings in the moment of solution, felt that the solution had come to them all at once, and were certain that their solution was correct. These three dimensions seem to be at the core of Aha! experiences, independent of solution correctness. However, although these three dimensions are shared, correctness is reflected in major quantitative differences between Aha! experiences that follow correct and incorrect solutions: Compared to incorrect, correct solutions were rated as more pleasant and more sudden and solvers were more confident about being correct. Further, a small qualitative difference was found: for correct solutions, Relief also uniquely predicted Aha! whereas for incorrect solutions, it was Surprise. This suggests a slightly different emotional coloring of the Aha! experience, with problem solvers who found the correct solution feeling relieved, and problem solvers who found an incorrect solution feeling surprised. Importantly, these differences were observed in the absence of any feedback about solution correctness. These findings speak against regarding the Aha! experience as only epiphenomenal (as for example suggested by Weisberg and Alba, 1981).

Looking at solution times, faster solutions were found to be more likely to be rated high on Aha! and slower solutions were more likely to be rated low, a result which is in accordance with several other studies (e.g., Aziz-Zadeh et al., 2009; Wegbreit et al., 2012; Chein and Weisberg, 2014; Danek et al., 2014b).

The results of the present study can be compared to the results of Webb et al.'s recent study (2016, reported in the same Research Topic). Although the motivation for the Webb et al. study was to explore how different dimensions underlying the Aha! experience might predict solution accuracy, and in contrast the motivation for the present study was to explore how different underlying dimensions might predict Aha! differently for correct and incorrect solutions, there are still a number of commonalities that can be noted across the results of the two studies. Differences between the two studies that might limit the comparability will be discussed later on, as well as unique insights that were gained from exploring relations for correct and incorrect solutions separately in the present study.

### Pleasure

There was a strong and positive relationship in simple correlations (*r* = 0.66) between Pleasure and the global feeling of Aha!. This finding seems to generalize across different problem solving tasks, with Webb et al. (2016) reporting *r*'s in the range of 0.71 to 0.73 when using five classic, mostly verbal insight problems and *r*'s ranging from 0.63 to 0.70 when using Compound Remote Associate (CRA) problems (Bowden and Jung-Beeman, 2003). It is also in accordance with another study on CRA problems by Kizilirmak et al. who report a more positive emotional response (measured on a 5-point graphical affective rating scale with smiley faces) for Aha! solutions compared to non-Aha! solutions (Kizilirmak et al., 2016b). It also matches our everyday experience of insight as a very pleasant event. Further, positive affect is known to facilitate insight (e.g., Isen et al., 1987; Bolte et al., 2003; Subramaniam et al., 2009; Sakaki and Niki, 2011). The present finding that feeling happy or in a good mood predicts a global rating of Aha! sheds some new light on these studies, at least on those where insight was assessed through self-reports. With positive emotions being a key aspect of the subjective Aha! experience, inducing positive mood prior to solving might simply lead participants to report more Aha! experiences. They may be more likely to say that any solution was an insight. This is in contrast to the hypothesis that being in a good mood increases the likelihood of insightful solutions (reflected in higher solving rates).

Another possible theoretical explanation for the prevailing role of Pleasure is offered by Thagard and Stewart's attempt to model the Aha! experience (Thagard and Stewart, 2011). Their EMOCON model conceptualizes the Aha! experience as a pattern of neural activity that arises through the convolution of an emotional reaction with a new combination of mental representations. Of course, a novel combination of representations (or restructuring) is just what is needed for solving a magic trick or other difficult problem solving tasks where solvers are lured into an inappropriate initial representation. The “ecstasy of discovery” (Thagard and Stewart, 2011, p. 10) is proposed to arise from automatic appraisal mechanisms that judge each new combination of mental representations with regard to its relevance. If the novel combination is non-trivial and highly relevant for the problem solver, a strong emotional response is triggered which is also reflected on a physiological level.

### Suddenness

The feeling that a solution appears all at once instead of stepwise was another unique predictor of Aha! in the present study, with a strong and positive simple correlation (*r* = 0.49) between Suddenness and the global Aha! rating. This means problem solvers who experienced the solution as very sudden were also likely to report a strong Aha! feeling. This supports the idea of different cognitive processing underlying solutions with stronger or weaker reported Aha! experiences. In the case of strong Aha! experiences, the solution pops into mind all at once, as a whole. Webb and colleagues did not gather data on this dimension, so it is unclear whether it might generalize across problem solving tasks. Further, perceived Suddenness depended on the degree of complexity of the solution, with single-step solutions feeling more sudden than multi-step solutions, independent of trick difficulty or time to solution.

Of course, because Suddenness was explicitly mentioned in the Aha! prompt that participants were given, that could be the reason for the strong relation between Suddenness and the global Aha! rating in this study. However, this simple explanation seems less likely when one considers that Suddenness was found to be more of a factor for tricks that required single-step solutions as opposed to multi-step solutions. This shows that there was not a simple positive relation between Suddenness and Aha! ratings which would be more consistent with a bias or demand characteristic resulting from Suddenness as being included as part of the Aha! prompt. It also highlights the importance of careful task analyses when selecting which problems to study, even with the recognition that any problem solving task can be solved with or without Aha! experience (Bowden et al., 2005; Öllinger et al., 2014; Kizilirmak et al., 2016a; Danek et al., 2016; Webb et al., 2016). Clearly, the aim for researchers who want to study insight and Aha! is to select tasks that not only have a high probability of leading to an initially biased problem representation which is false and must be improved through a representational change, but also to select tasks that have a high probability of triggering Aha! experiences. The present data indicates that mainly problems with single-step solutions will yield the feeling of Suddenness. This important new finding converges with a recent study on three classical insight problems (9 Dot, 8 Coin and one Matchstick Arithmetic Problem) reporting that problems with solutions for which only one constraint needs to be relaxed feel more like an “Aha!” than multi-step solutions with several constraints (Danek et al., 2016). The prototypical example of a multi-step solution problem is the classic 9 Dot Problem (Maier, 1930) which Kershaw and Ohlsson (2004) as well as Öllinger et al. (2014) have shown involves multiple causes of difficulty. These types of problems are not what insight researchers should aim for if they are trying to study Aha! experiences.

### Certainty

Confidence in the correctness of the proposed solution (in the absence of feedback) also uniquely predicted the strength of the global Aha! rating, with a simple correlation of *r* = 0.58 between Aha! and Certainty. Again, this finding seems to generalize across different problem solving tasks, with Webb et al. (2016) reporting *r*'s ranging from 0.60 to 0.65 (classic insight problems) and from 0.52 to 0.63 (CRAs). On one hand, the strong relation between Certainty and the global Aha! rating could be due to the fact that, like Suddenness, Confidence was stressed in the Aha! prompt that participants were given in both this study and the Webb et al. study (“You are relatively confident that your solution is correct without having to check it once more.”). However, other studies that have not included Certainty in their prompt (Hedne et al., 2016) have also found that Certainty is higher for Aha! trials than non-Aha! trials, which suggests that it may be an essential dimension of the Aha! experience even without explicit prompting.

### Relief

The affective dimension of Tension Release or Relief has not been widely explored previously. Webb et al. (2016) did include it by mentioning relief in the Aha! prompt, but did not collect data on it. Relief was found to be highly correlated with Aha! in this study (*r* = 0.49). The fact that it also correlated strongly with Pleasure (*r* = 0.64) suggests that the dimensions of Pleasure and Relief might be measuring similar emotional constructs. However, it is also possible that Relief is related to the cognitive process of representational change that allows the solver to resolve an impasse, overcome a difficulty, or escape fixation. Relief was the only dimension unique to correct solutions. This means, if a correct solution was found, problem solvers' Aha! ratings covaried with Relief ratings. This was not the case for incorrect solutions.

### Surprise

The overall relation between ratings on the Surprise dimension and Aha! was only 0.07 in simple correlations in this study, while the Webb study reports *r*'s ranging from 0.29 to 0.48 (classic insight problems) and from 0.15 to 0.25 (CRAs) for their Surprise dimension. There are a number of possible ways to interpret these differences. One possibility is that the Surprise ratings in the Webb study are capturing the same underlying perception as the Suddenness ratings in the present study, and our results turned out differently because we asked participants to rate both dimensions. Alternatively, because Webb et al. did not counterbalance the direction of their scales (all dimensions were aligned with the global Aha! rating), they may have inflated the positive relations among the dimensions. Of course, differences between the problem types (magic tricks vs. puzzles and CRAs) could also be responsible for differences in Aha! experiences, but this seems less likely given the high consistency with regard to the other dimensions.

Most importantly, the Surprise dimension was one of two dimensions (the other one was Relief) to suggest that Aha! experiences triggered by correct solutions slightly differ from those triggered by incorrect solutions, as the Surprise dimension was a unique predictor only for incorrect solutions. This result questions the wisdom of the established approach of using a multi-component operational definition for Aha! that encompasses Suddenness, Certainty and Surprise. Studies relying on Surprise in their Aha! prompts might actually have encouraged participants to use a misleading cue and therefore obtained invalid self-reports of insight.

### Drive

The overall relation between ratings on the Drive dimension and Aha! was 0.28 in this study (no Drive dimension was included in the Webb study). Interestingly, Drive was canceled out and did not predict the Aha! rating at all when variance due to subjects was removed (by fitting random intercepts for subjects in our mixed model analysis). These results suggest that Drive is just an individual factor that is experienced differently by each person, but that it is not a relevant part of the Aha! experience.

In future studies, it would be interesting to investigate possible cues problem solvers might be using for their subjective dimension ratings. For the dimension Suddenness, this study provides first evidence that solution complexity (single vs. multi-step solutions) plays a role in judging a solution as emerging suddenly or not. However, it remains unclear what leads problem solvers to feel that a solution is pleasant or relieving or surprising.

### Differences between the present study and Webb et al. (2016)

Comparing the present results with Webb et al.'s study (Webb et al., 2016) who used a very similar methodology on completely different problem sets offers the exciting possibility to scale the findings up to different tasks. This comparability might be a bit limited however, due to differences in the way the Aha! experience was assessed. Instead of a global Aha! rating like the one used here (“Did you have an Aha! moment?”, with a sliding scale from No to Yes), their “Aha” variable was measured as “Strength of the insight experience” (with a sliding scale from very weak to very strong). At first glance, this might seem like only a small difference, but in particular the lower end of the scale does not seem fully equivalent. The wording of the strength rating scale might suggest to participants that some form of insight always takes place, because the lowest possible rating would still mean “a very weak insight experience.” Thus, there is no room for “no Aha's”, only for weak Aha's. Similarly, the ratings for the underlying dimensions were not counterbalanced for their direction, meaning that they were always aligned with the Aha! rating. This may have inflated both ratings of Aha! and the relation between Aha! and each dimension if some subjects simply had a leftward or rightward bias when using the scales and might also explain why Webb et al. tended to find slightly higher correlations. Yet, despite these differences a number of commonalities were found.

In contrast to the present study, Webb et al. (2016) did not analyze incorrect and correct solutions separately. This makes sense given that the aim of Webb et al. (2016) was not to decompose the Aha! experience, but was instead to predict solution accuracy from the individual dimensions as well as from a global measure of Aha! (strength of the insight experience). However, the fact that differences were seen in the present study in which dimensions served as unique predictors of Aha! for correct and incorrect solutions shows that it is important to consider these different solution types separately. Several unique insights that emerged from exploring relations for correct and incorrect solutions separately included a better understanding of the Surprise dimension and its relation to both Aha! experiences and solution accuracy. Webb et al. found a consistently positive relationship between Surprise and Aha! which led them to conclude that Surprise is an important factor in the Aha! experience. At the same time, they reported a negative or non-significant correlation between Surprise and accuracy across three experiments and in their powerful multilevel regression model (combining data from 674 subjects), they found that Surprise decreased solution accuracy. This suggests a disconnect between the way Surprise relates to Aha! experiences and accuracy. By splitting solutions based on their correctness, in the present analysis it becomes clear that the relation between Surprise and Aha! may be specific for incorrect solutions. In other words, feelings of Surprise that accompany a solution may relate more strongly to false insights rather than true ones. Finally, the analysis for only correct solutions reveals Relief as the one dimension that relates more to correct than incorrect solutions, suggesting a slightly different emotional coloring of the Aha! experience, dependent on solution correctness.

## Conclusion

In sum, this study reports three main findings: First, false insights exist. Second, the Aha! experience is truly multidimensional, centered around both affect (Pleasure) and cognition (evaluating solutions as emerging suddenly and feeling confident about them). Third, although Aha! experiences for correct and incorrect solutions share these three common dimensions, they are also experienced somewhat differently with regard to magnitude and quality. Correct solutions emerged faster and led to stronger Aha! experiences; higher ratings of Pleasure, Suddenness, and Certainty; and were more associated with Relief, while incorrect solutions were more associated with Surprise.

Taken as a whole, these results cast some doubt on the assumption that the occurrence of an Aha! experience can serve as a definitive signal that a true insight has taken place. Theoretically, this would have suggested that Aha! experiences should have only resulted from correct solutions. Although the present study measured a rather comprehensive set of six dimensions, more work is needed to determine if there may be other specific aspects of the Aha! experience that may be more indicative of only true insights. Moreover, if we adopt the Gestalt psychologists' original definition of insight as being based on restructuring (Wertheimer, 1925), future studies should try to include some measure of restructuring. On the other hand, the quantitative and qualitative differences in the experience of correct and incorrect solutions demonstrate that the Aha! experience is not a mere epiphenomenon. To conclude, strong Aha! experiences are clearly, but not exclusively linked to correct solutions, and consist of three key components: joy of discovery, confidence in being correct and a feeling that the solution appears all at once.

## Ethics statement

This study was carried out in accordance with the recommendations of the Institutional Review Board and the Office for the Protection of Research Subjects of the University of Illinois at Chicago with written informed consent from all subjects. All subjects gave written informed consent in accordance with the Declaration of Helsinki. The protocol was approved by the Institutional Review Board and the Office for the Protection of Research Subjects of the University of Illinois at Chicago.

## Author contributions

AD and JW designed the experiment. AD developed the magic trick material, conducted the study and wrote the first draft of the manuscript. AD and JW analyzed the data. Both authors were critically involved in the interpretation of the results and in revising the manuscript.

## Funding

This work was funded by a grant to AD from the DFG (German Research Foundation), grant # DA 1683/1-1. The Research Open Access Publishing (ROAAP) Fund of the University of Illinois at Chicago provided financial support toward the open access publishing fee for this article.

### Conflict of interest statement

The authors declare that the research was conducted in the absence of any commercial or financial relationships that could be construed as a potential conflict of interest.

## Footnotes

^1^On average, solvers were over-confident on tricks they solved incorrectly, and under-confident on tricks they solved correctly. Because the majority of tricks were solved incorrectly, participants were on average 23.22% over-confident.

## Supplementary material

The dataset of the present study will be made available at the open repository for psychology data “PsychData” (https://www.psychdata.de/index.php?main=none&sub=none&lang=eng).
