# Supplementary material for: What about False Insights? Deconstructing the Aha! Experience along Its Multiple Dimensions for Correct and Incorrect Solutions Separately
Source: Front Psychol. 2017 Jan 20;7:2077. doi: 10.3389/fpsyg.2016.02077 (PMC5247466; doi:10.3389/fpsyg.2016.02077)
Supplement: Supplementary file 1 [file DataSheet1.docx]

**Appendix**

|  | **Trick Name** | **Magic Effect** | **Trick Description** | **Duration (in sec)** | **Solution Type** |
| --- | --- | --- | --- | --- | --- |
| 1 | Appearing Silk* | Appearance | A red silk handkerchief appears from nowhere | 6.32 | SS |
| 2 | Ball to Cube* | Transformation | A ball gets transformed into a cube | 13.24 | SS |
| 3 | Billiard Balls* | Appearance | A little red ball multiplies | 15.88 | SS |
| 4 | Bottled Scarf | Vanish | A red scarf disappears from a closed bottle | 26.32 | MS |
| 5 | Bouncing Egg | Physical impossibility | A real egg is bounced repeatedly on the floor without breaking | 24.76 | SS |
| 6 | Bowling Ball | Topological impossibility (size) | A large bowling ball is carried in a thin suitcase | 13.36 | SS |
| 7 | Box Shuffling | Appearance | Cards are shuffled, then suddenly the card box appears, with all the cards falling out of it | 18.0 | MS |
| 8 | Card Levitation^†^ | Telekinesis (Levitation) | A card “climbs” out of a deck of cards by no visible aid | 18.0 | MS |
| 9 | Cigarette | Vanish | Cigarette and lighter disappear while the magician tries to light his cigarette | 15.04 | SS |
| 10 | Coat Hanger* | Topological impossibility (size) | A wooden coat hanger is pulled out of a small purse | 23.32 | SS |
| 11 | Colour Changing Card 1* | Transformation | A card changes colour after being tossed into the air | 8.0 | SS |
| 12 | Colour Changing Card 2 | Transformation | The front card in a glass changes its colour after being covered by a silk handkerchief | 9.44 | SS |
| 13 | Floating Ball | Telekinesis (Levitation) | A ball is floating between the magician’s hands | 17 | SS |
| 14 | Floating Bun | Telekinesis (Levitation) | A bun is covered by a napkin and starts to float | 29.16 | SS |
| 15 | Han Ping Chien | Transposition | 3 coins wander from one hand into the other | 26.36 | MS |
| 16 | Ketchup Bottle* | Vanish | A ketchup bottle is put in a bag and disappears | 17.68 | SS |
| 17 | Linking Cards | Penetration | Cards are chained to each other and unchained without damage | 72.52 | MS |
| 18 | Match through Match* | Penetration | One matchstick wanders through another one without breaking it | 12.56 | SS |
| 19 | Orange to Apple* | Transformation | An orange is transformed into an apple | 10.16 | SS |
| 20 | Paper to Money* | Transformation | Sheets of white paper turn into 50 Euro bills | 28.56 | MS |
| 21 | Pen through Banknote* | Penetration | A banknote is pierced by a pen, but remains intact | 25.32 | SS |
| 22 | Penetrating Coin | Penetration | A coin penetrates a sealed glass | 25.48 | MS |
| 23 | Rope | Restoration | A rope is cut in two pieces and restored to one | 21.64 | SS |
| 24 | Rubik's Cube* | Transformation | An unsolved Rubik's cube is solved after being tossed into the air | 17.68 | SS |
| 25 | Salt | Vanish | Salt is poured in the fist from where it disappears | 18.64 | MS |
| 26 | Salt 'n Pepper | Vanish | Salt and pepper are poured into one hand and the pepper disappears | 32.6 | SS |
| 27 | Shuffled / Unshuffled* | Telekinesis | Cards are seen mixed face-up/face-down, before all facing the same way (as if they had turned over by themselves) | 42.52 | MS |
| 28 | Silk to Egg* | Transformation | A silk handkerchief turns into an egg | 20.6 | MS |
| 29 | Spoon | Transformation | A spoon is put into the magician's mouth and when removed, it has changed into a fork | 17.24 | SS |
| 30 | Three Card Monte* | Transposition | A card swaps places with another one | 38.28 | SS |
| 31 | Torn and Restored Card* | Restoration | A card is ripped in pieces and restored | 33.8 | MS |
| 32 | Vanishing and Appearing Coin^†^ | Appearance and Vanish | A coin is held up in the air, vanishes and reappears | 15.48 | SS |
| 33 | Vanishing Coin | Vanish | Out of three coins, one vanishes | 13.24 | SS |
| 34 | Vanishing Glass* | Vanish | A champagne glass is covered by a silk handkerchief and disappears | 9.84 | MS |
| 35 | Wandering Coin | Transposition | A coin wanders from the hand under a napkin | 19.04 | SS |
| 36 | Wandering Knives* | Transposition | Two differently coloured knives change places | 29.76 | SS |
| 37 | Water to Ice* | Transformation | Water is poured into a mug and transformed into an ice cube | 14.48 | SS |

MS = multi-step solution. SS = single-step solution. The tricks described here were already used in previous studies (e.g., Danek et al., 2013). Note that this list is not identical to the previous one (provided in Danek et al., 2014b) because we added three tricks to the set (#7, 8, 11) and modified some of the trick names to match the terminology in Hedne et al.’s study (2016) which is reported in the same Research Topic.

* This or a similar magic trick was also used by Hedne et al. (2016). The presentation of the trick as well as the method used to achieve the magic effect might be different.

^†^ This magic trick was excluded from the analyses because no participant provided a correct response.
